# Longing for Integrated Care: The Importance of Effective Governance

**DOI:** 10.5334/ijic.3510

**Published:** 2017-10-02

**Authors:** Mirella M.N. Minkman

**Affiliations:** 1 Tilburg University/TIAS School for Business and Society, NL; 2 Vilans National Center of Expertise in long term care, NL

**Keywords:** alignment of integrated care, collaborative governance, research agenda

Last March I had the honour to do my inaugural lecture at the University of Tilburg/TIAS Business School, where I have held since 2016 my chair called ‘Innovation of the organization and governance of integrated care’ [1]. For me it was a day to remember. In the Netherlands it is also a very formal and traditional ceremony where family, friends, colleagues and other relations are invited to share this moment. The inaugural lecture and the related book were a perfect reason for me to take some time for reflection on how to bring integrated care further and what challenges there are for a research agenda. This editorial is a pleasant invitation to share some of my ideas with the readers of our Journal.

My lecture was called ‘Longing for Integrated Care’ or in Dutch ‘Verlangen naar Integraliteit’. Of course this title was chosen for a reason. When I reflect on where we are in our way towards integrated care worldwide, I see that more and more clients, professionals and policy makers are looking in the direction of integrated care as a perspective; a perspective to reduce fragmentation because the real needs of people are often not really being seen and served. What really matters for a person like Mrs Van der Munt and her family, an 84 year old lady, living alone at home, becoming more and more fragile and heavily relying on her daughter to keep the promise that she can stay and die in her own home? What is the real issue in diabetes care? (Self) managing blood sugar levels? Or is it managing having diabetes in your social life, your cooking habits and daily living? Integrated care starts with a holistic perspective on what matters to people; otherwise the real essence of integrated care can be missed [1].

Integrated care is not about creating a multidisciplinary offer/supply, but it is about creating an integrative answer to the most important issues of people in need. A holistic approach seems logical, but it means a lot for how we organise our (health)care and welfare systems, and the needed connections with other domains in life [2]. Also, it asks for effective collaboration between professionals, clients and organizations. That also means a mis-fit with traditional governance which is mostly focusing on expanding or maintaining organizations or is professionally driven. Accountability is mostly targeted at ‘those who pay and those who can punish’ like health care insurers, policy makers and health care inspectorates. I expect that the era in which being mostly accountable towards clients, the community and the society will be on the rise.

## Growing interest in integrated care

Interest in integrated care is growing. This is reflected in the rising numbers of scientific publications and is manifest in both IJIC’s increased impact factor and the increasing number of participants at IFIC’s international conferences (over 1000 persons at ICIC17 in Dublin earlier this year). It is also stimulating to see that organizations like the World Health Organisation are developing conceptual frameworks that embrace integrated care like the Global Framework for People-centred and Integrated Health Services [3,4]. The need for interconnectedness and really adding value towards lives is also a broader development in our changing societies. The possibilities of the digital revolution increase our opportunities to connect, exchange and interact worldwide. Innovations like e-health and blockchain could have a major impact on stakeholders in health care systems, for instance on health care insurers as the way we exchange value changes.

At the same time we know that integrated care does not become reality automatically. Its development takes a long timeframe. Differences between countries are huge in the way they think about (health)care, their traditions, urgencies and political issues. That can be conflicting with ambitions like ‘implementing good practices as fast as possible’ and with the pressure to deliver results in politically set tight time frames.

## Governance of integrated care

For future directions, the governance of integrated care and interorganisational collaboration on a local level needs more attention, more innovative thinking and more knowledge. I define governance as the total package of leadership, accountability and supervision in the local setting in an area or region [5]. In my country, the Netherlands, a huge number of professional networks, for instance for dementia care, stroke care, elderly care or palliative care, have been set up in the last decade. An inventory among 135 of these networks showed that although in most cases the collaboration is signed up in a collaborative agreement between involved providers, commitment towards the network is not automatically present. Organizational interests predominate and often one of the partners is dominant in the network that may often result in reduced levels of cooperation [1].

Although the aim of these networks is to serve clients better, in one-third of them the interests of clients are not taken into account (according to the networks themselves). If clients are involved this is often via the professionals. Overall, the inventory shows that these networks explore and also struggle with how to organise integrated care and on what scale (population, target group, geographical area) [1]. Traditional governance *within* organisations often does not match the needed governance *between* organisations. Network governance is more horizontal, non-hierarchic, and focuses on trust as a basic value. In my view, the quality of the relationship between involved people and organizations could be a crucial factor to focus on in this type of governance [5,6].

In my opinion, future research should not only focus on interventions, costs and outcomes of integrated care programmes and cases, but also on how to organise effective (local) governance that encompasses people’s integrative needs and perspectives. Research topics in this field could include shared decision-making, trust, leadership, ownership and accountability of and to clients themselves [7]. Maybe *collaborative governance*, in which the focus is on the process in which (policy) goals are collaboratively worked on by the involved actors and clients/citizens, could gain new insights [8]. In this perspective more knowledge about the principles and values that lie underneath ways of working and collaborative governance are important to explore. This relates to the work of IFIC’s Special Interest Group on principles and values of integrated care [9]. I would like to encourage readers and writers to submit papers on these issues and invite you all to visit ICIC 2018 in Utrecht the Netherlands to further discuss these important topics.

## Alignment of integrated care

If we really want to achieve the ‘next level’ of integrated care then the way forward is to realize alignment between all of these components of integrated care: of vision (holistic perspective), organization and governance. If we do not manage to align these levels and free ourselves from thinking in terms like primary care, secondary care and tertiary care or other professional and organisational silos, then we will end up with suboptimal results. Alignment of integrated care on all levels is the future direction to proceed. I admit this is complex and not yet achieved, and countries remain in various stages of development due to competing priorities and interests. Therefore, integrated care will require continued long-term efforts, but is definitely worth longing for.
